# Late‐life time‐restricted feeding and exercise differentially alter healthspan in obesity

**DOI:** 10.1111/acel.12966

**Published:** 2019-05-21

**Authors:** Marissa J. Schafer, Daniel L. Mazula, Ashley K. Brown, Thomas A. White, Elizabeth Atkinson, Vesselina M. Pearsall, Zaira Aversa, Grace C. Verzosa, Leslie A. Smith, Aleksey Matveyenko, Jordan D. Miller, Nathan K. LeBrasseur

**Affiliations:** ^1^ Robert and Arlene Kogod Center on Aging Mayo Clinic Rochester Minnesota; ^2^ Department of Physical Medicine and Rehabilitation Mayo Clinic Rochester Minnesota; ^3^ Division of Biomedical Statistics and Informatics, Department of Health Sciences Research Mayo Clinic Rochester Minnesota; ^4^ Department of Surgery Mayo Clinic Rochester Minnesota; ^5^ Department of Physiology and Biomedical Engineering Mayo Clinic Rochester Minnesota

**Keywords:** aging, exercise, healthspan, obesity, physical activity, time‐restricted feeding

## Abstract

Aging and obesity increase multimorbidity and disability risk, and determining interventions for reversing healthspan decline is a critical public health priority. Exercise and time‐restricted feeding (TRF) benefit multiple health parameters when initiated in early life, but their efficacy and safety when initiated at older ages are uncertain. Here, we tested the effects of exercise versus TRF in diet‐induced obese, aged mice from 20 to 24 months of age. We characterized healthspan across key domains: body composition, physical, metabolic, and cardiovascular function, activity of daily living (ADL) behavior, and pathology. We demonstrate that both exercise and TRF improved aspects of body composition. Exercise uniquely benefited physical function, and TRF uniquely benefited metabolism, ADL behavior, and circulating indicators of liver pathology. No adverse outcomes were observed in exercised mice, but in contrast, lean mass and cardiovascular maladaptations were observed following TRF. Through a composite index of benefits and risks, we conclude the net healthspan benefits afforded by exercise are more favorable than those of TRF. Extrapolating to obese older adults, exercise is a safe and effective option for healthspan improvement, but additional comprehensive studies are warranted before recommending TRF.

## 1 INTRODUCTION

The number of overweight and obese older adults is steadily increasing. Of Americans age 65 or older, more than 62% have a body mass index (BMI) ≥25 and 30% have a BMI ≥ 30 (Kalish, 2016). Aging and obesity dramatically increase the risk for an array of chronic and comorbid conditions that truncate healthspan, the period of disease‐ and disability‐free life. Devising effective and safe solutions to health challenges arising from the convergence of aging and obesity is, therefore, a decisive priority. The multifaceted consequences of obesity at older ages have highlighted the need for interventions that target fundamental metabolic and aging pathways, under the hypothesis that targeting central mechanism may provide multisystem benefit. While several pharmacological approaches are being pursued (Longo et al., 2015), physical exercise and dietary interventions unequivocally improve the health of multiple organ systems, at least in young mammals.

Long‐term aerobic exercise training enhances body composition and reduces the risk of cardiovascular disease, cancer, dementia, and physical frailty, among other chronic conditions (Pedersen & Saltin, 2006). Similarly, time‐restricted feeding (TRF), which is ad libitum food access limited to an 8‐ to 12‐hr period daily, robustly reprograms metabolic physiology. In young mice, TRF beneficially impacts a range of health parameters including diurnal rhythm, body composition, glucose tolerance, insulin resistance, hepatic metabolism, and aspects of inflammation (Chaix, Zarrinpar, Miu, & Panda, 2014; Delahaye et al., 2018; Hatori et al., 2012; Sherman et al., 2012; Sundaram & Yan, 2016). Although metabolic enhancements have also been observed in middle‐aged mice (Chaix et al., 2014; Duncan et al., 2016), we are unaware of any studies that have tested whether aged, obese mice experience healthspan benefits through TRF, and to our knowledge, cardiovascular function following TRF has not been explored in young or aged mice. Similarly, while we and many others have extensively explored the effects of exercise in young and middle‐aged mice (Schafer et al., 2016), far fewer studies have comprehensively examined the health effects of a late‐life exercise intervention in obese mice. It is possible that benefits identified in youth may not unanimously translate to older ages, given that declines in systemic plasticity and metabolic flexibility accompany aging.

Here, we performed a comparative effectiveness study of voluntary running wheel exercise versus TRF on multiple parameters of healthspan in aged mice with diet‐induced obesity. We focused our healthspan profiling on phenotype domains strongly linked to obesity and aging, namely body composition, physical and cardiovascular function, ADL behavior, metabolism, and pathology. We demonstrate that exercise and TRF, even when initiated in late‐life, are distinctly able to improve multiple domains of healthspan in obese mice, including body composition, metabolism, physical function, ADL behavior, and liver pathology. Critically, TRF adversely affects both lean mass and vascular function. Consequently, our parallel assessments of healthspan in aging and obesity indicate that exercise and TRF confer overlapping, yet distinct benefits. Our findings confirm the safety of exercise, but elicit caution and warrant further study of TRF as an intervention to improve late‐life health.

## 2 RESULTS

### 2.1 TRF and exercise remodel body composition

**Figure 1:**
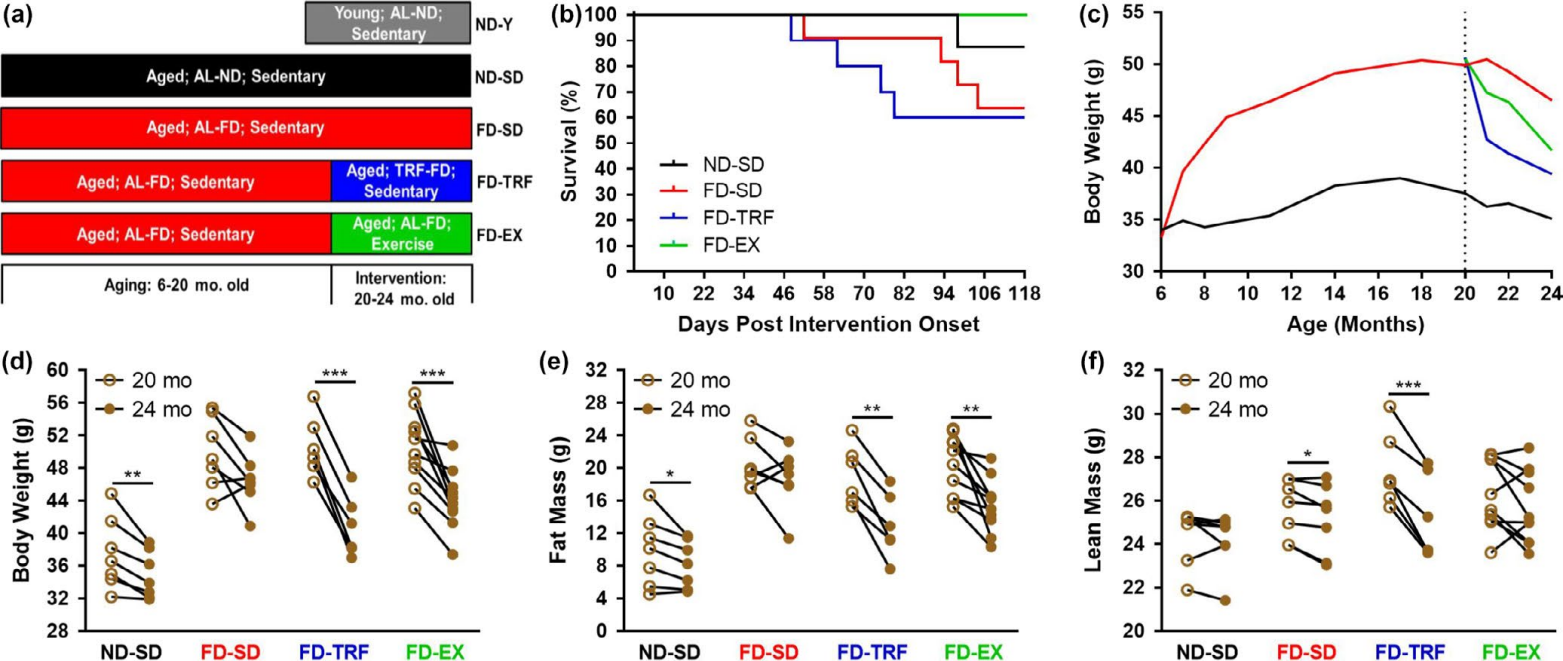
Time‐restricted feeding and exercise uniquely influence survival and body composition in aged, obese mice. (a) Schematic description of experimental mouse groups. (b) Mortality of aged mice during the 4‐month intervention phase. (c) Longitudinal mean body weight throughout the entire study. Total (d) body, (e) fat, and (f) lean mass in grams (g) at intervention onset (age 20 months; open circles) and endpoint (age 24 months, filled circles) are depicted (paired *t*‐tests) (*n* = 6–10) (*p* < *0.05,**0.01,***0.005) (ND‐SD = old, sedentary, ad libitum (AL) normal diet; FD‐SD = old, sedentary, AL fast‐food diet; FD‐TRF = old, sedentary, 8 hr dark cycle AL access to fast‐food diet; FD‐EX = old, voluntary running wheels, AL fast‐food diet)

To comparatively study the healthspan benefits conferred by aerobic exercise versus TRF in aged, obese mice, we began by administering 6‐month‐old mice a standard chow diet (normal diet [ND]) or fast‐food diet (FD), enriched with saturated fat, cholesterol, and fructose. At 20 months of age, a time point approximated to be a human equivalent age of 60 years, mice on the FD were randomized to: continue ad libitum FD with sedentary lifestyle (FD‐SD), receive ad libitum FD limited daily to an 8‐hr period of the dark cycle with sedentary lifestyle (FD‐TRF), or ad libitum FD with 24‐hr access to running wheels (FD‐EX; Figure 1a). Mice were maintained on the interventions for 4 months and were euthanized at 24 months of age, a time point approximated to be a human equivalent age of 70 years. Average daily energy intake from food and high fructose water measured over a 12‐week period during the 4‐month intervention period did not statistically differ among aged mouse groups (Figure S1A). Body weight normalized energy intake, however, was slightly lower in FD‐SD mice, compared to ND‐SD (Figure S1B), which is consistent with prior observations in young sedentary, ad libitum‐fed ND and FD mice (Hatori et al., 2012). FD‐EX mice ran 1.3 mean and 0.7 median kilometers per day. A group of 6‐month‐old sedentary mice fed ad libitum ND (ND‐Y) was also assessed at endpoint to determine aging effects that were obesity‐ and intervention‐independent. All mice were healthspan phenotyped in the month preceding necropsy. Nine mice died during the 4‐month interventional window prior to necropsy: 1/8 ND‐SD (13%), 4/11 FD‐SD (36%), and 4/10 FD‐TRF (40%). No premature mortality occurred in the FD‐EX group (*n* = 10) (Figure 1b), corresponding to a trend toward improved survival for exercised mice, relative to FD‐SD mice (Fischer's exact test: *p* = 0.0902). Healthspan data correspond only to mice euthanized at endpoint.

At 20 months of age and prior to randomization, mice maintained on the FD weighed 34% more (Figure 1c) and were comprised of 56% more fat mass and 20% less lean mass, relative to ND‐SD mice. During the 4‐month intervention, aged mice in all experimental groups underwent significant changes in body composition. ND‐SD mice lost an average of 3 g or 7% of their body weight (Figure 1d), which corresponded to a significant reduction in fat mass (Figure 1e). A trend toward reduced body weight (*p* = 0.0562) was identified in FD‐SD mice (Figure 1d), which corresponded to a significant reduction in lean mass (Figure 1f). FD‐TRF lost an average of 10 g or 20% of their body weight, and FD‐EX lost an average of 6 g or 12% of body weight (Figure 1d). Both TRF and exercised mice lost an average of 6 g of fat mass (Figure 1e). FD‐EX mice maintained relatively stable lean mass during the 4‐month intervention window, but FD‐TRF mice lost an average of 2 g of lean mass (Figure 1f). Following intervention onset, FD‐TRF mice that died spontaneously were not able to stabilize their body mass within a week following diet introduction (Figure S2A). This outcome was not associated with anorexic behavior, since TRF mice that died prematurely consumed average energy equivalent to those that survived until necropsy (Figure S2B). Accordingly, we speculate that maladaptive catabolic processes may have occurred in aged FD‐TRF mice, which may have been dependent on impaired ability to maintain lean mass. Conversely, exercised mice maintained lean mass and had reduced total body fat.

### 2.2 TRF and exercise differentially alter physical function and nesting behavior

**Figure 2:**
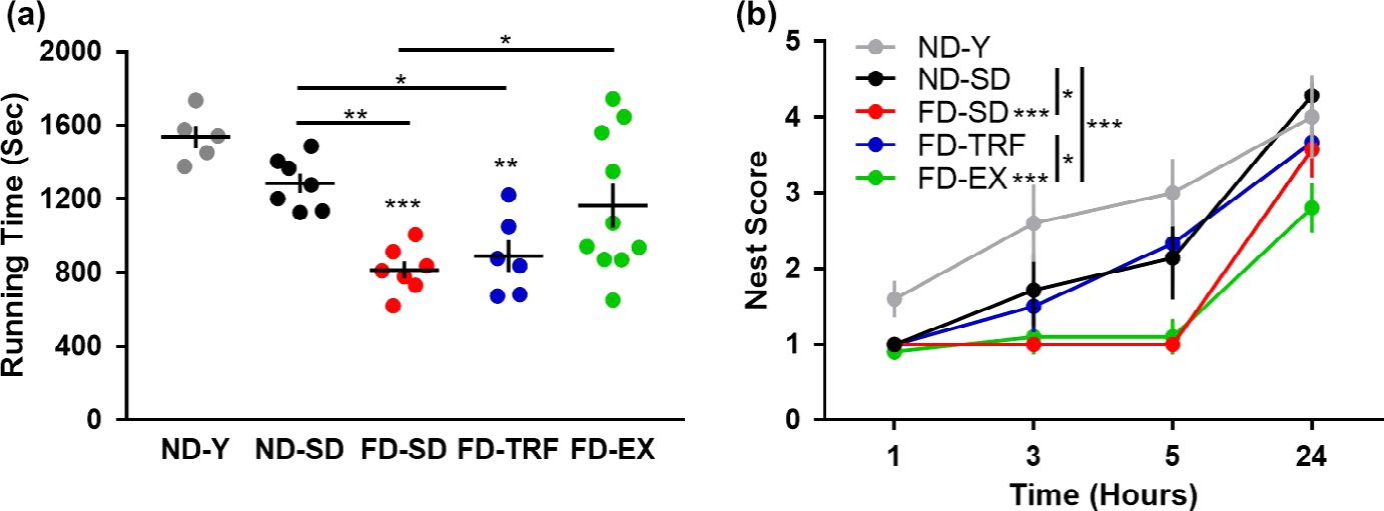
Exercise improves physical function and time‐restricted feeding improves nesting behavior in aged, obese mice. (a) Duration ran to exhaustion was assessed on a motorized treadmill test (ANOVA). (b) Nest quality was assessed at 1, 3, 5, and 24 hr in an activity of daily living behavioral test (two‐way ANOVA) (*n* = 5–10) (Mean ± *SEM*) (*p* < *0.05,**0.01,***0.005) (ND‐Y = young, sedentary, ad libitum normal diet; ND‐SD = old, sedentary, ad libitum normal diet; FD‐SD = old, sedentary, ad libitum fast‐food diet; FD‐TRF = old, sedentary, 8‐hr dark cycle ad libitum access to fast‐food diet; FD‐EX = old, voluntary running wheels, ad libitum fast‐food diet)

We next tested the effects of the interventions in the context of aging and obesity on physical function and nest building performance. Physical function, a key parameter of healthspan decline in humans and rodents, was measured by the duration mice ran to exhaustion on a motorized treadmill. Diet‐induced obesity significantly exacerbated the shift in age‐dependent physical decline, with FD‐SD mice running significantly shorter durations, relative to ND‐Y and ND‐SD mice (Figure 2a). Exercise improved this outcome, with FD‐EX mice running significantly greater mean durations, relative to FD‐SD mice. TRF did not improve physical function, as measured by the treadmill test.

Nest building is an ADL task that models executive and personality changes often observed in older adults with cognitive decline (Deacon, 2006). We observed a non‐significant reduction in nest building performance as a function of normal aging in ND‐SD, relative to ND‐Y mice (Figure 2b). Obesity exacerbated the age‐related change, with FD‐SD mice producing nests of poorer quality than those of ND‐Y or ND‐SD mice. The obesity effect was improved by TRF, but exercise did not improve nest building performance.

### 2.3 TRF alters metabolic function

**Figure 3:**
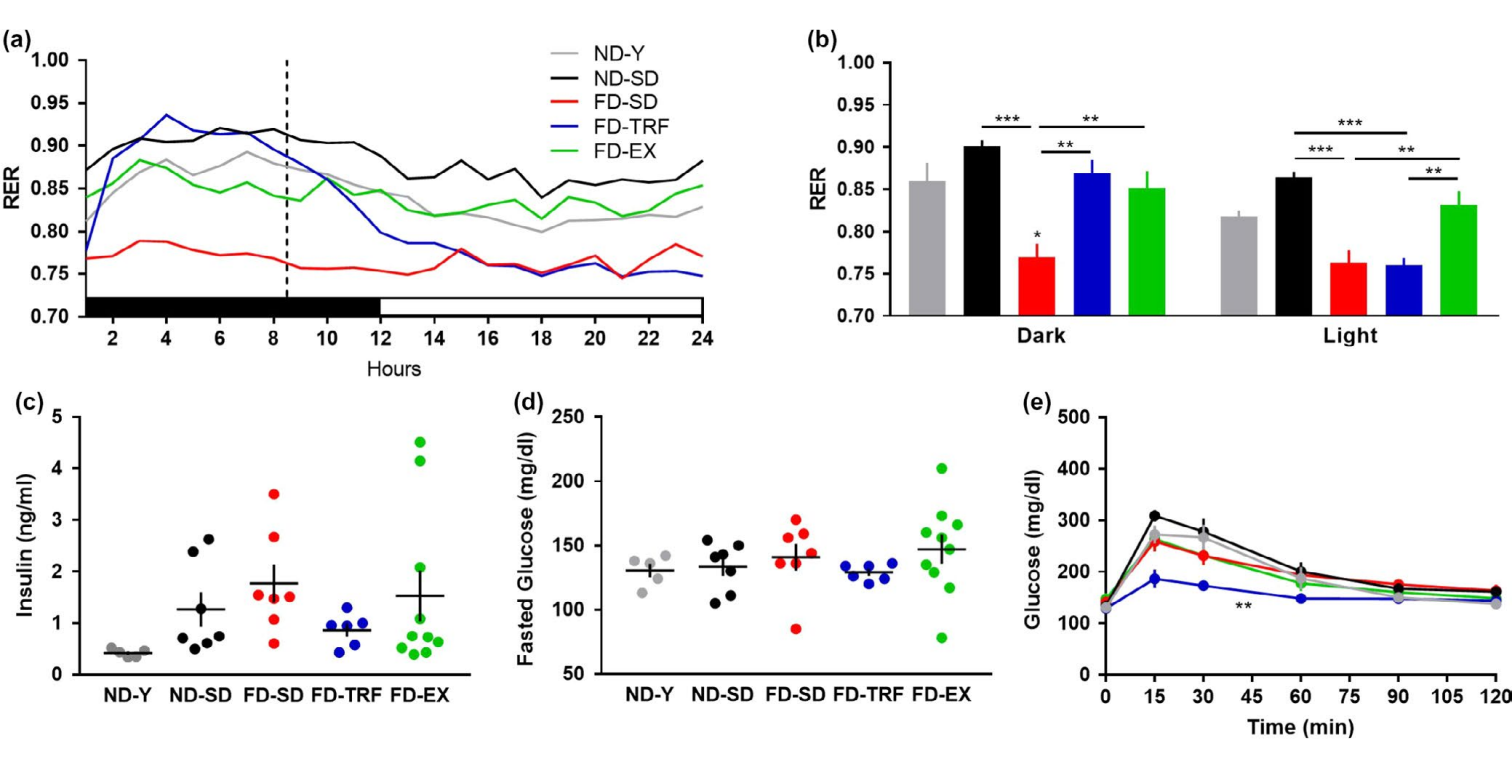
Time‐restricted feeding alters metabolism. (a) Average respiratory exchange ratios (RER) over one 12‐hr dark (black bar), 12‐hr light (white bar) cycle are indicated. FD‐TRF mice received food and high fructose water during the first 0.5–8.5 hr of the dark cycle (dotted line indicates food removal). All other groups received food ad libitum. (b) Quantification reflecting total mean RERs during the dark and light cycles (ANOVA per phase). (c) Insulin levels in nonfasted plasma were assessed at endpoint (ANOVA). (d) Fasted glucose levels were assessed at the beginning of a (e) GTT, in which 1.25 g/kg glucose was administered intraperitoneally, and circulating glucose levels were measured 15, 30, 60, 90, and 120 min later (two‐way ANOVA) (*n* = 5–10) (Mean ± *SEM*) (*p* < *0.05,**0.01,***0.005) (ND‐Y = young, sedentary, ad libitum normal diet; ND‐SD = old, sedentary, ad libitum normal diet; FD‐SD = old, sedentary, ad libitum fast‐food diet; FD‐TRF = old, sedentary, 8‐hr dark cycle ad libitum access to fast‐food diet; FD‐EX = old, voluntary running wheels, ad libitum fast‐food diet)

To assess metabolic function, we began by implementing whole‐body calorimetry. As indicated by respiratory exchange ratio (RER), ND‐fed mice displayed natural diurnal rhythms, reflective of greater metabolic activity during the dark cycle (Figure 3a,b). FD‐SD mice displayed an absence of RER cyclicity, maintaining consistently low RERs throughout both the dark and light cycle. TRF dramatically altered RER rhythm, with FD‐TRF mice exhibiting elevated RERs during the dark cycle, suggestive of higher carbohydrate utilization. RERs of FD‐TRF mice dropped precipitously following fasting onset, suggestive of a shift to higher fat utilization. Relative to TRF, exercise had a reduced effect on RER cyclicity; however, FD‐EX mice maintained RER levels that were similar to ND‐Y mice, indicative of potential benefit.

Assessment of insulin levels and glucose tolerance testing (GTT) revealed additional metabolic consequences. Although not significant using multiple comparison testing, aging and obesity appeared to adversely influence nonfasted blood insulin levels (Figure 3c). TRF appeared to dampen the obesity‐ and aging‐dependent increase, while the effect of exercise was more heterogeneous. Fasted glucose levels did not statistically differ between groups (Figure 3d). A GTT revealed striking enhancement of glucose tolerance in FD‐TRF mice (Figure 3e). Metabolic adaptations induced by TRF likely correspond to regular fasting rather than reduced energy intake, since average energy intake did not statistically differ among the aged, FD experimental groups (Figure S1).

### 2.4 TRF, but not exercise, attenuates cardiovascular function

**Figure 4:**
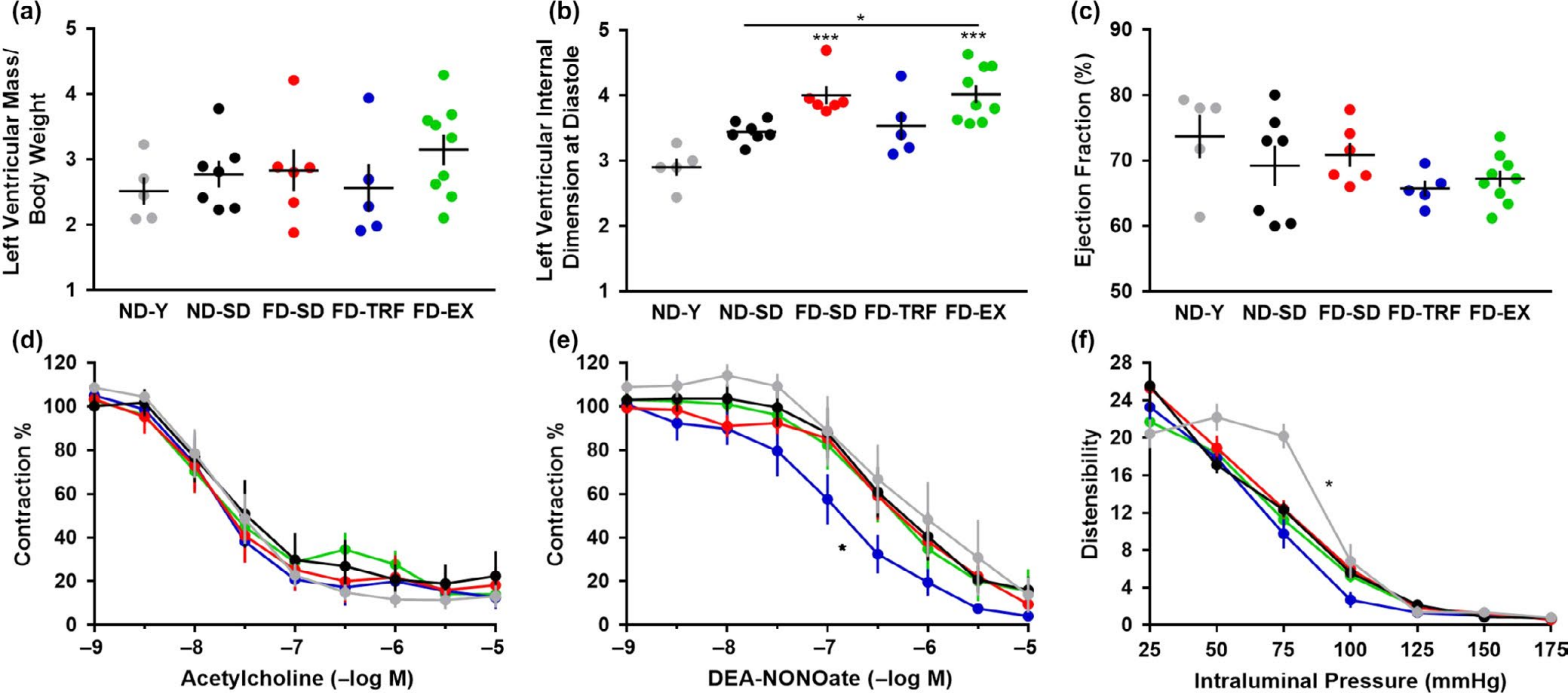
Time‐restricted feeding influences cardiovascular function. Echocardiography was used to assess (a) left ventricular mass (normalized to body weight), (b) left ventricular internal dimensions at end‐diastole, and (c) left ventricular ejection fraction (ANOVA). Isolated carotid artery segments were assessed in a pressurized organ chamber bath system to determine changes in vessel diameter in response to (d) acetylcholine, (e) DEA‐NONOate, and (f) passive pressure (two‐way ANOVA) (*n* = 5–10) (Mean ± *SEM*) (*p* < *0.05,**0.01,***0.005) (ND‐Y = young, sedentary, ad libitum normal diet; ND‐SD = old, sedentary, ad libitum normal diet; FD‐SD = old, sedentary, ad libitum fast‐food diet; FD‐TRF = old, sedentary, 8‐hr dark cycle ad libitum access to fast‐food diet; FD‐EX = old, voluntary running wheels, ad libitum fast‐food diet)

We next tested whether the interventions altered age‐ or obesity‐related cardiovascular parameters. Neither heart weight normalized to body weight (data not shown) nor body weight adjusted left ventricular mass (Figure 4a) were significantly altered by aging, obesity, or the interventions, although a slight elevation was observed in FD‐EX mice. Aging and obesity, however, increased left ventricular internal dimension at diastole (Figure 4b), and the obesity‐dependent increase was attenuated in FD‐TRF mice, but not FD‐EX mice. It is important to note that structural and dimensional changes in FD‐EX may reflect activity‐dependent physiological adaptations that are independent of adverse, obesity‐dependent adaptations. Despite alterations in operating dimensions, ejection fraction was preserved in FD‐SD mice (Figure 4c).

Isolated, pressurized carotid arteries were examined for functional vascular changes. We observed no statistically significant age‐, obesity‐, or intervention‐dependent alterations in relaxation to acetylcholine, suggestive of preserved endothelial function across experimental groups (Figure 4d). Similarly, no differences in vascular relaxation to DEA‐NONOate were observed when comparing ND‐Y, ND‐SD, FD‐SD, and FD‐EX, but FD‐TRF mice displayed augmented relaxation, reflecting a greater sensitivity to nitric oxide (Figure 4e). Passive pressure assessments in calcium‐free conditions demonstrated age‐associated reductions in carotid distensibility (Figure 4f). At physiologic pressures, comparison of aged, FD groups indicated that FD‐TRF mice displayed significantly reduced distensibility, reflective of increased vascular stiffness (Table 1).

**Table 1:** Healthspan index: Benefits and risks of TRF and exercise in aged, obese mice

	Summary data: Mean ± *SEM* or Number (%)			Effect: Positive = 1, None = 0, Negative = −1		*p*‐Value	
	FD‐SD	FD‐TRF	FD‐EX	FD‐TRF	FD‐EX	FD‐TRF	FD‐EX
Reduced fat mass	20 months: 20.5 ± 1.2 g 24 months: 18.7 ± 1.4 g	20 months: 19.2 ± 1.5 g 24 months: 13.0 ± 1.6 g	20 months: 20.5 ± 1.2 g 24 months: 15.4 ± 1.0 g	1	1	0.0095^a^	0.0461^a^
Maintained lean mass	20 months: 25.6 ± 0.5 g 24 months: 25.2 ± 0.6 g	20 months: 27.5 ± 0.7 g 24 months: 25.2 ± 0.8 g	20 months: 26.3 ± 0.5 g 24 months: 25.7 ± 0.5 g	−1	1	0.0197^a^	0.8349^a^
Increased running time	814.6 ± 47.3 s	890.8 ± 87.3 s	1,165.0 ± 120.3 s	0	1	0.8474^b^	0.0396^b^
Enhanced nest building performance	AUC: 47.9 ± 3.5	AUC: 63.8 ± 5.9	AUC: 41.7 ± 4.9	1	0	0.0266^c^	0.5142^c^
Improved glucose tolerance	AUC: 23,691 ± 1,418	AUC: 18,692 ± 404.9	AUC: 22,645 ± 1,579	1	0	<0.0001^c^	0.6563^c^
Reduced circulating insulin	1.8 ± 0.4 ng/ml	0.9 ± 0.1 ng/ml	1.5 ± 0.5 ng/ml	0	0	0.3116^b^	0.8852^b^
Increased ejection fraction	70.9 ± 1.8%	65.7 ± 1.2%	67.2 ± 1.3%	0	0	0.0716^b^	0.1509^b^
Increased carotid distensibility	AUC: 1,335 ± 96.5	AUC: 1,116 ± 71.4	AUC: 1,229 ± 47.2	−1	0	0.0195^c^	0.1436^c^
Reduced liver enzymes (either)	ALP: 100.3 ± 13.1 U/L ALT: 125.6 ± 13.6 U/L	ALP: 47.5 ± 8.5 U/L ALT: 96.2 ± 21.4 U/L	ALP: 69.4 ± 11.2 U/L ALT: 106.3 ± 16.5 U/L	1	0	ALP: 0.0148^b^ ALT: 0.4503^b^	ALP: 0.1158^b^ ALT: 0.6332^b^
Reduced tumor burden	5/7 (71%)	3/6 (50%)	7/10 (70%)	0	0	0.5921^d^	>0.9999^d^
Total				2	3		

a ANOVA of 24‐month values, adjusting for baseline 20‐month values; model includes FD‐SD, FD‐EX, and FD‐TRF, with FD‐SD as control.

b ANOVA of 24‐month values; model includes FD‐SD, FD‐EX, and FD‐TRF, with FD‐SD as control.

c 2‐way ANOVA including FD‐SD, FD‐EX, and FD‐TRF, with FD‐SD as control.

d Fisher's exact test.

### 2.5 TRF and exercise influence morbidity parameters

**Figure 5:**
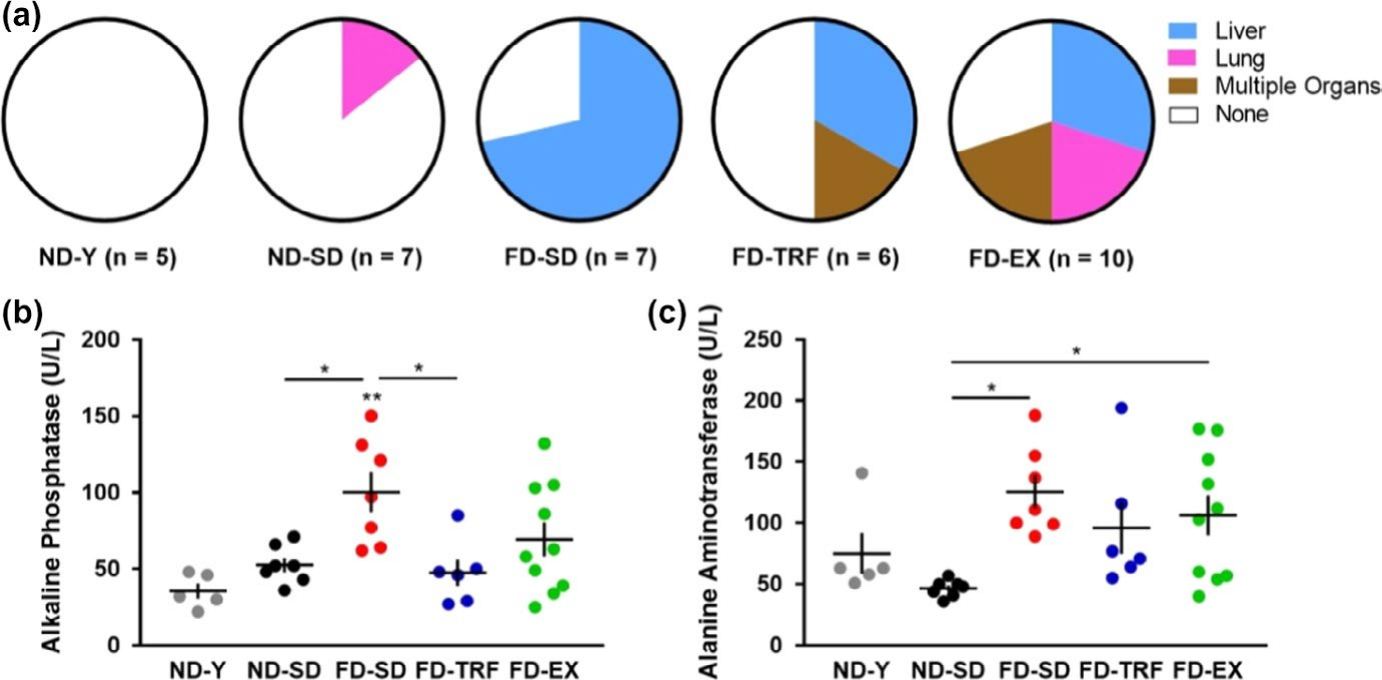
Effect of exercise and time‐restricted feeding on tumor burden and circulating biomarkers of liver health. (a) Evidence of gross pathological changes consistent with tumor burden was assessed in all experimental groups. Circulating levels of liver enzymes (b) alkaline phosphatase and (c) alanine aminotransferase were analyzed in blood collected at endpoint (ANOVA) (*n* = 5–10) (Mean ± *SEM*) (*p* < *0.05,**0.01,***0.005) (ND‐Y = young, sedentary, ad libitum normal diet; ND‐SD = old, sedentary, ad libitum normal diet; FD‐SD = old, sedentary, ad libitum fast‐food diet; FD‐TRF = old, sedentary, 8‐hr dark cycle ad libitum access to fast‐food diet; FD‐EX = old, voluntary running wheels, ad libitum fast‐food diet)

Cancer and liver pathology are common in aged, obese mice. Thus, we assessed tumor prevalence and levels of circulating liver enzymes at endpoint. Aging and obesity robustly influenced tumor‐associated morbidity. No tumors were identified in ND‐Y mice, 1/7 ND‐SD mice (14%) were tumor positive, and 5/7 FD‐SD mice (71%) were tumor positive (Figure 5a). Relative to FD‐SD, TRF and exercise did not significantly alter cancer prevalence (Table 1); 3/6 FD‐TRF mice (50%) and 7/10 FD‐EX mice (70%) were tumor positive (Figure 5a).

Assessment of liver enzymes as surrogate biomarkers of liver health revealed a striking obesity effect. Both alkaline phosphatase (ALP) and alanine aminotransferase (ALT) were significantly elevated in FD‐SD mice, relative to ND‐SD mice (Figure 5b,c). FD‐TRF significantly reduced ALP levels and dampened ALT levels. The effect of FD‐EX on circulating ALP and ALT was heterogeneous. FD‐EX and FD‐TRF mice also had significantly reduced mean liver weights normalized to body weight, relative to FD‐SD mice (data not shown).

### 2.6 Exercise and TRF confer net healthspan benefits, but TRF also confers adverse effects

Since TRF and exercise had distinct effects on individual healthspan parameters, we devised an index to compare the outcomes of each intervention across domains in an unbiased manner. We selected key functional parameters corresponding to body composition, physical function, ADL task performance, metabolism, cardiovascular function, and morbidity and assessed individual benefits for each parameter by comparing the effects of TRF and exercise, relative to aged, obese controls (comparison groups: FD‐SD, FD‐EX, and FD‐TRF; Table 1). TRF benefited four parameters: fat mass, nest building performance, glucose tolerance, and liver pathology (circulating ALP levels). Exercise benefited three parameters: fat mass, lean mass, and physical function. Critically, TRF caused two adverse outcomes: loss of lean mass and reduced vascular distensibility. FD‐EX mice did not experience any adverse outcomes. Upon account of detrimental effects, the composite healthspan index of the FD‐EX was greater than the index of FD‐TRF mice (Table 1).

## 3 DISCUSSION

Healthspan decline with advancing age reflects dysfunction across one or multiple physiologic systems and is accelerated in the context of obesity. Population aging and the high burden of obesity in older adults emphasize the need for safe and effective approaches to mitigate age‐related disease and disability. Physical exercise and TRF are behavioral interventions that robustly improve obesity‐related health parameters at young and middle ages, but their comparative benefits and risks in aged, obese organisms, until now, were unknown. Here, we report the first study testing the effects of TRF versus exercise on the healthspan of aged, obese mice.

We demonstrate that initiation of TRF or exercise in late‐life improves multiple, yet distinct, healthspan parameters in aged, obese mice. Specifically, exercise and TRF both reduced body fat, but exercise alone led to preservation of lean mass. This is a critical finding, since lean mass is an important mediator of physical function, whole‐body metabolism, and organismal resilience (Huffman, Schafer, & LeBrasseur, 2016; St‐Onge, 2005). Prior studies have reported preservation of lean mass following TRF in younger mice (Chaix et al., 2014; Hatori et al., 2012), indicating that this detrimental outcome may be unique to aged organisms and further highlighting the import of this finding. We also show that FD‐TRF mice that were not able to body weight stabilize in the week following intervention onset died spontaneously, suggesting reduced adaptive potential in older mammals may be related to lean mass maintenance. Exercised, obese, aged (FD‐EX) mice maintained lean mass and exhibited 100% survival. FD‐EX mice also had improvements in physical function, as measured by the duration ran to exhaustion in a treadmill test. Increased running duration was not observed in TRF mice that lost lean mass, further highlighting the association between lean mass maintenance and functional performance. Recent randomized clinical trials of exercise interventions in older adults with clear evidence of functional decline have shown that exercise can enhance function and prevent future disability (Pahor et al., 2014). Importantly, the robust effects of exercise on lean mass and physical function, especially when combined with weight loss, are conserved in older adults burdened by obesity (Villareal et al., 2011).

Independence in ADL tasks is another important indicator of healthy aging. TRF uniquely enhanced nest building performance, an ADL measure in mice relevant to human health. Moreover, the effect of TRF on measures of metabolism was greater than that of exercise and in accordance with previously published findings in younger mice with diet‐induced obesity (Chaix et al., 2014; Hatori et al., 2012). Our data demonstrating metabolic flexibility in the form of altered RER diurnal rhythm, improved glucose tolerance, and normalized liver enzymes emphasize the potent effect of daily fasting in the absence of total calorie restriction, even in late‐life, on metabolic health.

In addition to the benefits of exercise on multiple domains of health in older adults, the safety of exercise is widely recognized. In aged, obese mice, we did not observe any adverse exercise‐mediated outcomes and, interestingly, mice randomly assigned to the exercise intervention exhibited a trend toward greater survival, independent of an effect on tumor burden. In contrast, TRF adversely affected key health outcomes that caution against its immediate application in overweight, older humans. Specifically, TRF mice experienced loss of lean mass and reductions in vascular distensibility. To our knowledge, cardiovascular outcomes tested here have not been examined in prior studies of therapeutic TRF interventions in young or aged mice, but experiments in *Drosophila* have demonstrated that TRF suppresses aspects of age‐ and diet‐dependent cardiac dysfunction (Gill, Le, Melkani, & Panda, 2015). Thus, further investigation of the effects of TRF on mammalian cardiovascular function throughout the lifespan is warranted.

Our findings build on considerable work in young mice and lay the ground work for exploration into the tissue‐specific mechanisms by which exercise and TRF benefit or adversely affect aged, obese mice. Work in young mice has implicated coordinated nutrient sensing and circadian pathways as primary mediators of beneficial physiologic TRF adaptations (Chaix et al., 2014; Hatori et al., 2012) and are expected to be similarly regulated in aged mice, although potentially in a diminished capacity. We found that aged mice demonstrate dampened physiologic adaptivity, relative to previous findings in young mice (Chaix et al., 2014; Hatori et al., 2012; Schafer et al., 2016). The mechanisms underlying diminished physiologic flexibility in response to intervention throughout the lifespan are also unknown, although reduced tissue plasticity and epigenetic patterning are candidate mediators (Hanley et al., 2010; Rao & Mattson, 2001). Furthermore, the mechanisms responsible for detrimental cardiovascular outcomes following TRF are unknown. Food restriction has been previously associated with reprogramming of stress pathways (Pankevich, Teegarden, Hedin, Jensen, & Bale, 2010), which could drive maladaptive cardiovascular remodeling, including vascular stiffness (Goodson et al., 2017). Indeed, running wheel exercise is voluntary and may reduce stress, while TRF is investigator‐imposed and may increase stress. Feeding time is an important determinant of circulating stress hormone levels (Ahlers, Smajda, & Ahlersova, 1980), but in young mice, limited temporal profiling suggests TRF may reduce, rather than raise, corticosterone levels (Chaix et al., 2014). Whether aberrant stress signaling in aged, obese mice may underlie adverse cardiovascular remodeling is an important unanswered question.

Obesity, particularly in the context of aging, strongly increases risk for remodeling body composition, adversely affecting physical function and behavior, and developing metabolic dysfunction, cardiovascular disease, fatty liver disease, and cancer, which is why these healthspan outcomes were the focus of our study. It will be important in future interventional aging studies to expand healthspan profiling to additional functional domains that may also be altered, including immune, gastrointestinal and microbiome, skeletal, and respiratory systems. Testing of combined or modified intervention paradigms, such as TRF plus voluntary exercise or gradually imposed TRF, which may maintain or enhance benefits but alleviate adverse outcomes, will also be of great value.

In summary, our results demonstrate that similar to humans, murine aging is characterized by a consistent decline in healthspan reflecting dysfunction in multiple organ systems with diet‐induced obesity exacerbating healthspan decline. Both voluntary physical exercise and TRF improve distinct healthspan parameters, but the negative effects of TRF on lean mass and vascular function may offset any benefit to overall organismal healthspan and signal caution with respect to its application to older adults burdened by obesity. Thus, the utility of TRF as a practical behavioral intervention for overweight or obese older adults may be limited, but efficacy and safety results support aerobic exercise as a translatable approach for reversing healthspan decline.

## 4 EXPERIMENTAL PROCEDURES

### 4.1 Experimental model

Male C57/BL6 mice harboring a senescence‐related transgenic construct (Baker et al., 2011) were used for these studies. At 6 months of age, group‐housed mice were randomized to ad libitum access to normal diet (ND) (13% energy as fat, PicoLab Rodent Diet 20 (5053), Lab Diet) or a fast‐food diet (FD) (40% energy as fat (milk fat, 12% saturated) with 0.2% cholesterol (Western Diet (5342), Test Diet) with high fructose in the drinking water (42 g/L) (Charlton et al., 2011; Schafer et al., 2016), based on birthdate, body weight, and body composition using quantitative magnetic resonance (EchoMRI‐100; Akasaki et al., 2013). For the following 14 months, all mice were maintained on a sedentary lifestyle. At 20 months of age, body composition was assessed and FD mice were randomized to continue a sedentary lifestyle with ad libitum, 24‐hr FD access (FD‐SD), receive unlimited access to voluntary running wheels with 24‐hr ad libitum FD access (FD‐EX), or sedentary lifestyle with ad libitum FD access restricted to only 8 hr of the dark cycle (FD‐TRF). For FD‐EX mice, voluntary exercise behavior was monitored using Wheel Manager Data Acquisition Software (Med Associates). To control for enrichment, locked running wheels were randomized and rotated through all nonexercise groups. FD‐TRF mice were provided food 30 min following the start of the dark cycle. At the end of the 8‐hr feeding interval, FD‐TRF mice were switched to a cage free of food and high fructose water to ensure no food was available. All ad libitum (non‐TRF) mice were handled at the beginning and end of the FD‐TRF 8‐hr food access window, to control for extra handling in the TRF group. Throughout the entire study, all mice were provided ad libitum access to water. Food and high fructose water intake were monitored over 13 weeks to estimate average daily caloric intake. All mice in the study were maintained on the same 12‐hr light, 12‐hr dark cycle, which they were adapted to beginning 2 weeks before the intervention phase. For the intervention portion of the study, all mice were individually housed. Mice were monitored daily during the intervention period for survival. Three‐month postintervention onset, FD‐SD, FD‐EX, FD‐TRF, the control ND mice maintained on a sedentary lifestyle (ND‐SD), and a control cohort of 6‐month‐old sedentary male mice with 24‐hr ad libitum ND access (ND‐Y) were healthspan phenotyped. Mice were handled daily and transported to procedure areas at least 1 hr before each procedure to reduce stress. Mice were singly applied to all procedures, which were conducted in a blinded manner with thorough cleaning between each mouse. All healthspan phenotyping was conducted during the dark phase, with the exception of whole‐body calorimetry and nest building testing, which were conducted over 24‐hr periods. Mice were euthanized 4‐months postintervention onset. Experiments were performed under protocols approved by the Mayo Clinic IACUC.

### 4.2 Body composition and organ assessments

Body weight was measured weekly. Body composition (total body lean and fat mass) was assessed at study baseline (prior to ND vs. FD randomization), at intervention baseline (prior to FD‐SD, FD‐EX, FD‐TRF randomization), and at study endpoint by quantitative magnetic resonance (EchoMRI‐100; Akasaki et al., 2013). At endpoint, mice were heparinized and euthanized with a lethal dose of pentobarbital. Fresh whole blood was applied to VetScan Comprehensive Diagnostic Profile rotors (Abaxis) for analysis of circulating alanine aminotransferase and alkaline phosphatase. Carotid arteries were isolated for dynamic structure analysis as described following. Tumor burden was assessed by two research scientists who were blinded to the study groups. Organs were immediately dissected and weighed.

### 4.3 Metabolic function

A Comprehensive Lab Animal Monitoring System (CLAMS) equipped with an Oxymax Open Circuit Calorimeter System (Columbus Instruments) was used to assess oxygen consumption (VO_2_) and carbon dioxide (VCO_2_) production of individual mice over a 24‐hr period (one dark and one light cycle) following 18 hr of habituation (LeBrasseur et al., 2009). VO_2_ and VCO_2_ values were used to calculate respiratory exchange ratio (RER). Circulating glucose levels and sensitivity were determined following a 6‐hr fast by measuring concentrations using blood glucose monitoring strips (Bayer) before (time 0) and 15, 30, 60, and 120 min after an intraperitoneal bolus of glucose (1.25 g/kg) (Bernardo et al., 2010). Nonfasted circulating insulin levels were determined by enzyme‐linked immunosorbent assay (ELISA; Alpco) from plasma obtained at necropsy.

### 4.4 Cardiovascular function

Cardiac function was evaluated in mice under light isoflurane anesthesia by echocardiography using the Vevo 2100 system (FUJIFILM VisualSonics, Inc.; Roos et al., 2013). At the level of the papillary muscles, cardiac function was assessed from parasternal short‐axis images. Vascular reactivity was evaluated using carotid artery segments dissected under a microscope (Zeiss) and sutured to pressurized microcannula chambers (Living Systems) containing 37°C, flowing (rate: 50 ml/min), aerated (95% O_2_, 5% CO_2_) Krebs‐Ringer bicarbonate solution (d'Uscio, Smith, & Katusic, 2001; Roos et al., 2016). The chamber was positioned on an inverted microscope (Nikon Diaphot‐TMD) with an amplified image transmitted to a monitor and video dimension analyzer for lumen diameter measurement. Following equilibration, a submaximal contraction to thromboxane analog 9,11‐dideoxy‐11α,9 α‐epoxymethanoprostaglandinF_2_ α (U46619; 3 x 10^−8^ to 10^−6^ mol/L) was applied to test responsivity. After a 30‐min washout, the following three protocols were applied with 30 min of washing and equilibration between each. (a) Endothelium‐dependent relaxation: U46619 was applied according to the test concentration dose to stimulate a 50% vessel contraction. Concentration‐dependent relaxation to acetylcholine (ACH; 10^−9^ to 10^−5^ mol/L) was determined, with 4‐min equilibration and lumen diameter measurement between each dose. (b) Endothelium‐independent relaxation: U46619 was applied to stimulate a 50% vessel contraction. Concentration‐dependent relaxation to diethylammonium(Z)‐1‐(N,N‐diethylamino)diazen‐1‐ium‐1,2‐diolate (DEA; 10^−9^ to 10^−5^ mol/L) was determined, with 4‐min equilibration and lumen diameter measurement between each dose. (c) Passive compliance: Transmural arterial pressure was reduced to 25 mmHg. Following a 45‐min equilibration with calcium‐free Krebs, pressure was increased from 25 to 200 mmHg by 25 mmHg steps, with 10‐min equilibration and lumen diameter measurement between each step. Analyses were terminated following a return to 25 mmHg, with final equilibration and lumen diameter measurement. For relaxation experiments, dose–response curves were generated based on the mean difference in diameter change relative to baseline per drug dose. For passive compliance experiments, cross‐sectional compliance was calculated as the change in luminal cross‐sectional area relative to change in intravascular pressure. Distensibility was calculated as compliance normalized for the luminal cross‐sectional area prior to a pressure increment. LabChart8 was used to generate dynamic arterial measurements.

### 4.5 Physical function

Physical function was assessed using a motorized treadmill test of exercise capacity (Columbus Instruments; LeBrasseur et al., 2009). Mice were acclimated to the treadmill for three consecutive days, immediately followed by a test day. Acclimation consisted of 5 min starting at a speed of 5 m per minute for 2 min, then 7 m per minute for 2 min, followed by 9 m per minute for 1 min, at an incline grade of 5%. On the test day, mice ran on the treadmill at a 5% grade and an initial speed of 5 m per minute for 2 min. Every subsequent 2 min, the speed was increased by 2 m per minute until the mice were exhausted. Exhaustion was defined as the inability to remain on the treadmill despite a mild electrical shock stimulus and gentle mechanical prodding. Running time and distance were recorded.

### 4.6 Nest building task

For nesting behavior evaluation, one cotton nestlet (Ancare) was placed on a clean cage floor prior to placing the mouse in the cage. Nest quality was scored after 1, 3, 5, and 24 hr according to an established scale of 0–5, based on the degree of nestlet shredding, whether the shredded pieces were gathered or spread throughout the cage, and how compact the nest was (Deacon, 2006).

### 4.7 Quantification and statistical analyses

GraphPad Prism 7.04 and R were used for statistical analysis and generation of graphs. Data are expressed as the mean ± *SEM*. *p* ≤ 0.05 was considered statistically significant. One‐way or two‐way ANOVA with post hoc multifactor comparison was used for the comparison of multiple groups. For longitudinal body composition data, paired *t*‐tests or ANOVA models adjusting for baseline values were used when appropriate. Fischer's exact tests were used to interrogate nonrandom associations between categorical variables with small sample sizes.

## CONFLICT OF INTEREST

None declared.

## AUTHOR CONTRIBUTIONS

MJS designed the study, collected and analyzed data, and drafted and revised the manuscript. DLM, AKB, TAW, VMP, GCV, LAS, and AM collected and analyzed data and reviewed the manuscript. EA provided statistical support. ZA helped design the study and reviewed the manuscript. JDM provided study resources, analyzed data, and revised the manuscript. NKL designed the study, provided study resources, analyzed data, and revised the manuscript.
