# Supplementary material for: Late‐life time‐restricted feeding and exercise differentially alter healthspan in obesity
Source: Aging Cell. 2019 May 21;18(4):e12966. doi: 10.1111/acel.12966 (PMC6612646; doi:10.1111/acel.12966)
Supplement: Supplementary file 1 [file ACEL-18-e12966-s001.docx]

**
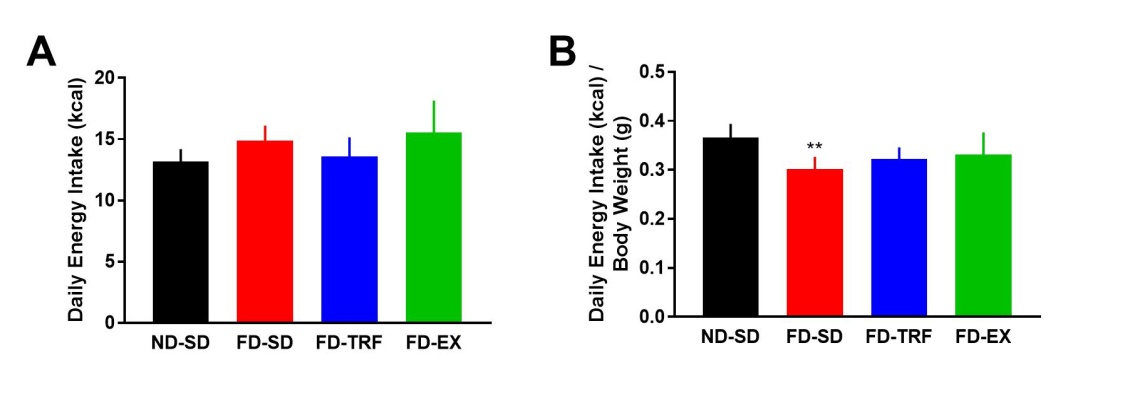
**

**Supplementary Figure 1. Energy consumption in aged mice.** (A) Average daily energy intake was estimated from weekly measurement of food and high fructose water consumption over 12 weeks, following the first week of diet/intervention, which served as an adaptive phase. (B) Daily energy consumption was normalized to body weight (ANOVA) (n = 5-10) (Mean ± SEM) (p < **0.01) (ND-SD = old, sedentary, ad libitum normal diet; FD-SD = old, sedentary, ad libitum fast-food diet; FD-TRF = old, sedentary, 8 hour dark cycle ad libitum access to fast-food diet; FD-EX = old, voluntary running wheels, ad libitum fast-food diet).

**
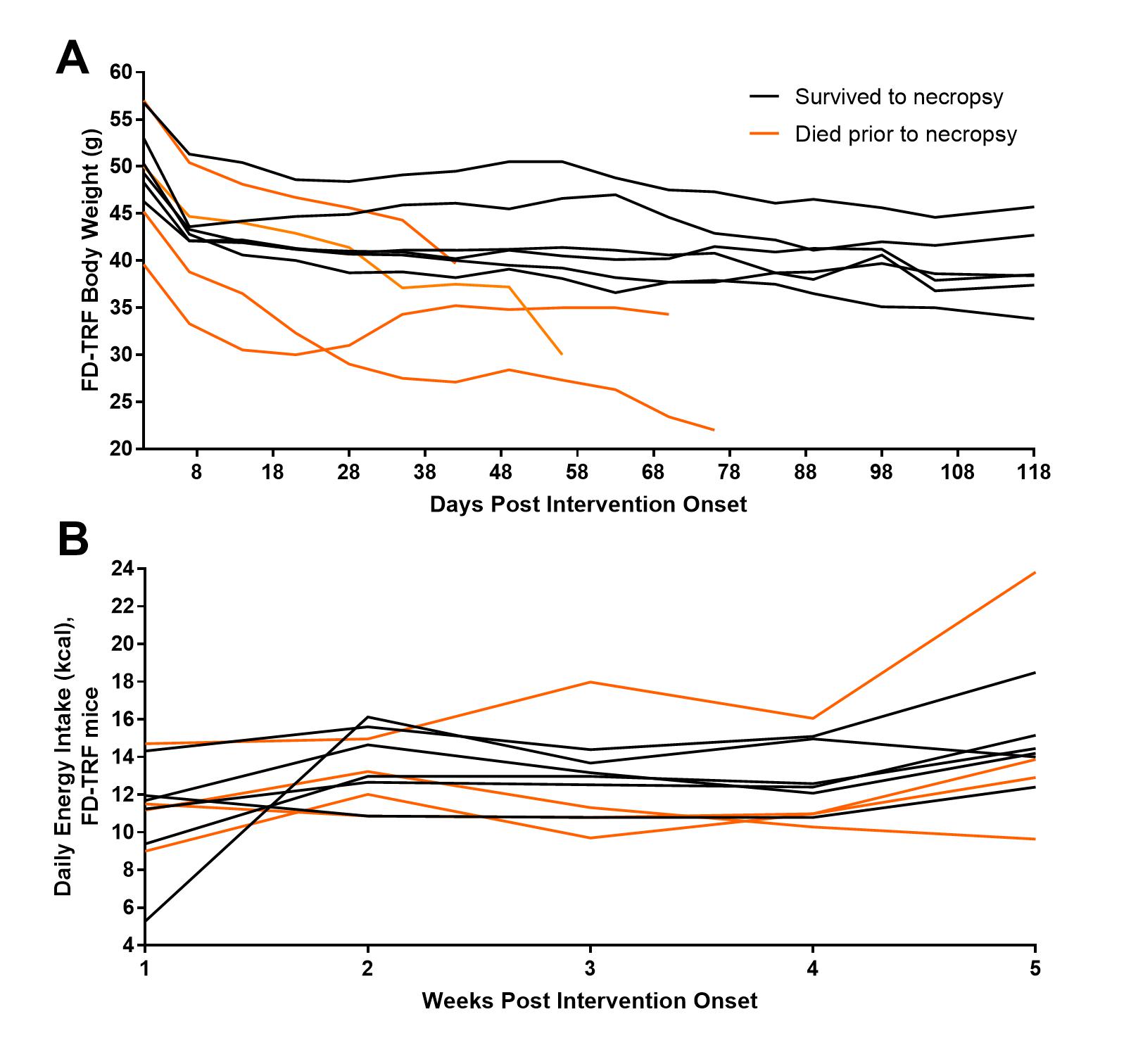
**

**Supplementary Figure 2. Inability to maintain body mass in the absence of anorexic behavior is associated with mortality in aged, obese TRF mice.** (A) Individual body weights of all mice randomized to FD-TRF are depicted, with mice that died spontaneously before necropsy indicated by orange lines. Inability to stabilize body weight within a week of TRF onset was identified in all mice that died prematurely on the TRF regimen. (B) Average daily energy intake estimates of all FD-TRF mice indicate that mice that expired spontaneously before necropsy (orange lines) consumed energy equivalent to TRF-counterparts that successfully adapted to the diet and survived to necropsy (black lines) (FD-TRF = old, sedentary, 8 hour dark cycle ad libitum access to fast-food diet).
